# Impact of Trace Mineral Source and Phytase Supplementation on Prececal Phytate Degradation and Mineral Digestibility, Bone Mineralization, and Tissue Gene Expression in Broiler Chickens

**DOI:** 10.1007/s12011-024-04076-w

**Published:** 2024-02-08

**Authors:** Hanna Philippi, Vera Sommerfeld, Alessandra Monteiro, Markus Rodehutscord, Oluyinka A. Olukosi

**Affiliations:** 1 Institute of Animal Science, University of Hohenheim, 70599 Stuttgart, Germany; 2 Animine, 74960 Annecy, France; 3 Department of Poultry Science, University of Georgia, Athens, GA 30602 USA

**Keywords:** Trace mineral source, Phytate degradation, Phytase, Bone mineralization

## Abstract

The objective of this study was to determine how different sources of Zn, Mn, and Cu in the feed without and with phytase affect prececal *myo*-inositol hexakisphosphate (InsP_6_) breakdown to *myo*-inositol (MI), prececal P digestibility, bone mineralization, and expression of mineral transporters in the jejunum of broiler chickens. A total of 896 male broiler chicks (Cobb 500) were distributed to 7 diets with 8 replicate pens (16 birds per floor pen). Experimental diets were fed from day 0 to 28. Diets were without or with phytase supplementation (0 or 750 FTU/kg) and were supplemented with three different trace mineral sources (TMS: sulfates, oxides, or chelates) containing 100 mg/kg Zn, 100 mg/kg Mn, and 125 mg/kg Cu. Prececal InsP_6_ disappearance and P digestibility were affected by interaction (phytase × TMS: *P* ≤ 0.010). In diets without phytase supplementation, prececal InsP_6_ disappearance and P digestibility were greater (*P* ≤ 0.001) in birds fed chelated minerals than in birds fed sulfates or oxides. However, no differences were observed between TMS in diets with phytase supplementation. Ileal MI concentration was increased by exogenous phytase but differed depending on TMS (phytase × TMS: *P* ≤ 0.050). Tibia ash concentration as well as Zn and Mn concentration in tibia ash were increased by phytase supplementation (*P* < 0.010), but the Cu concentration in tibia ash was not (*P* > 0.050). Gene expression of the assayed mineral transporters in the jejunum was not affected by diet (*P* > 0.050), except for *Zn transporter 5* (phytase × TMS: *P* = 0.024). In conclusion, the tested TMS had minor effects on endogenous phytate degradation in the digestive tract of broiler chickens. However, in phytase-supplemented diets, the choice of TMS was not relevant to phytate degradation under the conditions of this study.

## Introduction

Trace minerals (TM) such as Zn, Mn, and Cu are essential in animal nutrition due to their involvement in hormone and enzymatic systems. They are also components of proteins and play a role in the immune system [1]. Consequently, TM impact growth, bone development, and enzyme structures, among other things. If their supply is below the requirement, adverse effects on bone development and growth can be observed [1, 2]. Because plant-based feedstuffs commonly used in broiler diets do not contain adequate concentrations of available Zn, Mn, and Cu for the bird, their diets are usually supplemented with exogenous sources of those TM. According to the National Research Council [3], supply recommendations for broilers are 40 mg/kg Zn, 60 mg/kg Mn, and 8 mg/kg Cu in complete diets. However, TM concentrations used by the industry are often higher because they are used to promote growth performance, as in the case of Cu [4], or because they are supplemented with large safety margins to account for dietary antagonists such as phytate. However, the oversupply of trace minerals leads to elevated concentrations of trace minerals in the manure and is potentially harmful to the environment.

Phytate is the salt form of phytic acid (*myo*-inositol 1,2,3,4,5,6-hexakisphosphate (InsP_6_)) and the primary P source in feedstuffs derived from plant seeds. In non-ruminant animals, the phytate-P availability is limited and varies among species [5]. Phosphatases and phytases (*myo*-inositol hexaphosphate phosphohydrolases) are required for the dephosphorylation of phytate. Although broilers can degrade relevant amounts of phytate from diets with low mineral P and Ca contents by endogenous mucosal and microbial phosphatases [6], industry-type poultry diets are commonly supplemented with exogenous phytases to increase the digestibility of phytate-P further.

In vitro studies have shown that divalent cations can inhibit the phosphate release from InsP_6_ by exogenous phytases depending on the concentration and source of (trace) minerals [7, 8]. The inhibition could be due to reduced accessibility of the phytate molecule, because divalent cations such as Zn, Mn, and Cu can form insoluble complexes with phytate [9, 10]. The affinity of cations to form complexes with phytate was ranked as follows: Cu^2+^ > Zn^2+^ > Co^2+^ > Mn^2+^ > Fe^3+^ > Ca^2+^ [11]. Different trace mineral sources (TMS) can differ in their dissolution behavior, which might affect their potential to interact with phytate, with faster dissolving sources having a greater potential to interact than slower dissolving sources [12]. Indeed, it was observed in vitro that Cu sulfate reduced phytate solubility and phosphate release to a greater extent than Cu oxide, which may be due to the faster dissolution of Cu sulfate as opposed to Cu oxide [4]. Chelated TMS may have a lower interaction potential than inorganic sources because chelated TM are bound to a ligand that may prevent the mineral from interactions with other nutrients such as phytate. Therefore, sulfates, oxides, and chelates were selected as TMS in the present study and analyzed for their interaction potential with phytate. In order to investigate potential interactions, InsP_6_ and its degradation products were analyzed and InsP_6_ disappearance was calculated.

The first aim of this study was to investigate how different sources of Zn, Mn, and Cu, at levels commonly used by the industry, affect prececal phytate degradation, ileal concentration of partially dephosphorylated inositol phosphates (InsP_x_) and *myo*-inositol (MI), and prececal P digestibility. The second aim was to investigate whether TMS and exogenous phytase affect trace mineral concentration in the bone, expression of mineral transporters in the jejunum, and expression of genes related to protein synthesis and degradation in the breast muscle and the liver. We hypothesized that first, the impairment of phytate degradation by Zn, Mn, and Cu supplementation depends on TMS, and second, phytase supplementation affects Zn, Mn, and Cu concentration in the bone.

## Materials and Methods

### Birds and Housing

The trial was conducted at the Poultry Science Research Complex of the University of Georgia (Athens, GA, USA) following the approved protocol (IACUC number: A2021-06–006) of the Institutional Animal Care and Use Committee of the University of Georgia. A total of 896 day-old male broilers (Cobb 500) were used in a 28-day experiment. Birds were allocated to 56 floor pens (16 birds per pen) with wood shavings. Even weight distribution across all treatments and blocks was ensured when placing the birds on day 0. Seven treatments were arranged in a randomized complete block design with 8 replicate pens per treatment. Throughout the trial, tap water and feed were provided for ad libitum consumption. The barn was continuously illuminated for the first 3 days; from day 3, the period of darkness was gradually increased to 8 h per day. The room temperature was continuously decreased from 34 to 22°C. Health status of the birds was monitored daily.

### Experimental Diets

Experimental diets were corn-soybean meal based (Table 1) and were fed in 2 phases, starter and grower. The starter diets were fed in mash form (day 0 to 12), and the grower diets were fed as pellets (3 mm diameter, day 12 to 28). Diets were formulated to meet or exceed all nutrient requirements according to the National Research Council [3] except non-phytate P (Table 3). In the grower diets, titanium dioxide was added as an indigestible marker (5 g/kg). In a 2 × 3 + 1 factorial arrangement, 7 experimental diets were prepared. The experimental factors were phytase supplementation (0 (PHY–) or 750 FTU/kg (PHY+) declared activity of an *E. coli*–derived 6-phytase (Quantum®Blue, AB Vista, UK) and TMS. The TMS was a combination of Zn, Mn, and Cu (100, 100, 125 mg/kg feed) supplemented either as hydrated sulfates (Eastern Minerals, Inc. Henderson, NC, USA), oxides (Animine, France), or chelates (Availa® (Availa Zn, 12%; Availa Mn, 8%; Availa Cu, 10%), Zinpro, MN, USA). Levels of Zn and Mn were chosen according to the recommendation of the breeding company [13]. The high levels of Cu were chosen to represent Cu levels used in non-European countries to promote growth performance [4]. A phytase-supplemented diet containing supplemented sulfates to reach the National Research Council [3] recommended levels of trace minerals (15 mg/kg Zn, 40 mg/kg Mn, 7.5 mg/kg Cu) was used as an additional treatment to test the effect of trace mineral concentration. The chosen phytase supplementation level of 750 FTU/kg allowed for a clear phytase effect while staying below the possible maximum InsP_6_ degradation. To prepare the starter diets, all non-variable ingredients were mixed to obtain a basal diet. The starter basal diet was divided into 7 parts and supplemented with 4 g/kg of the corresponding trace mineral premix (Table 2) and quantity of phytase. For the grower diets, a premix with all non-variable mineral ingredients, vitamins, and free amino acids was prepared and split into 7 parts. Corn, soybean meal, soybean oil, and premix were supplemented with 4 g/kg of the corresponding trace mineral premix (Table 2) and quantity of phytase. Since TM premixes differed in Ca concentrations, limestone was added accordingly on top to achieve the same Ca concentration in all treatments. Representative samples were taken for each treatment and ground through a 0.5-mm sieve for chemical analyses (Retsch, ZM 200, Haan, Germany).

**Table 1:** Ingredients of starter diet (day 0 to 12) and grower diet (day 12 to 28)

Ingredient, g/kg	Starter	Grower
Corn	585.9	634.7
Soybean meal	365.0	315.0
Soybean oil	15.5	17.0
TiO_2_	0.0	5.0
Dicalcium phosphate^1^	17.7	10.9
Limestone	1.6	2.9
Lysine (HCl)	0.8	1.2
DL-Methionine	2.5	2.4
Threonine	0.2	0.2
NaHCO_3_	2.0	2.0
Sodium chloride	3.0	3.0
Vitamin premix^2^	1.0	1.0
Choline	0.8	0.7
Individual trace mineral premix^3^	4.0	4.0

^1^18.5% P

^2^Vitamin premix (DSM, Netherlands), provided per kg of complete feed: 3527 IU vitamin A, 1400 IU vitamin D3, 19.4 IU vitamin E, 0.01-mg vitamin B12, 1.1-mg menadione, 3.5-mg riboflavin, 5.5-mg pantothenic acid, 1.0-mg thiamine, 20.3-mg niacin, 1.5-mg vitamin B6, 0.6-mg folic acid, 0.1-mg biotin

^3^Individual trace mineral premixes were manufactured to contain individual concentrations of Zn, Mn, and Cu from different sources (see Table 2)

**Table 2:** Trace mineral premix composition in mg/kg complete feed

Minerals	Sulfates	Oxides	Chelates	Low-sulfates
Zn	100	100	100	15
Mn	100	100	100	40
Cu	125	125	125	7.5
Mg as Mg oxide	100			
Fe as Fe sulfate	40			
Se as Na selenite	0.35			
I as Ca iodate	1			

Differences between calculated and analyzed Zn and Mn concentrations did not exceed 22% (Table 3). For Cu, differences did not exceed 25%, and for Ca, the maximal deviation was 12%. Differences between calculated and analyzed P concentrations did not exceed 10%. Maximal deviation of InsP_6_-P concentration did not exceed 20%. The maximal deviation of calculated and analyzed phytase activity values was 14% in the starter phase and 21% in the grower phase.

**Table 3:** Calculated and analyzed composition of the experimental starter and grower diets

Treatment		P	InsP_6_-P	Ca	Zn	Mn	Cu	Fe	Phytase activity
		g/kg DM			mg/kg DM				FTU/kg
		(Starter) Calculated composition							
Phytase, FTU/kg	TMS								
0	Sulfates	8.1	3.1	8.1	146	134	148	126	< 50
0	Oxides								< 50
0	Chelates								< 50
750	Sulfates								750
750	Oxides								750
750	Chelates								750
750	*Low-sulfates*	8.1	3.1	8.1	51	66	16	126	750
Analyzed composition (Starter)									
0	Sulfates	7.6	n. a	8.5	154	163	157	138	< 50
0	Oxides	7.6		8.4	160	141	135	147	< 50
0	Chelates	7.9		9.1	174	158	144	158	< 50
750	Sulfates	7.8		8.6	151	112	139	155	660
750	Oxides	8.3		9.4	165	144	154	165	683
750	Chelates	7.7		8.7	187	156	159	159	815
750	*Low-sulfates*	7.6		8.9	64	63	21	170	832
		Calculated composition (Grower)							
0	Sulfates	6.4	3.0	6.7	145	131	148	119	< 50
0	Oxides								< 50
0	Chelates								< 50
750	Sulfates								750
750	Oxides								750
750	Chelates								750
750	*Low-sulfates*	6.4	3.0	6.7	49	64	16	119	750
Analyzed composition (Grower)									
0	Sulfates	6.4	2.8	7.6	139	111	116	148	< 50
0	Oxides	6.2	2.7	6.8	145	113	120	113	< 50
0	Chelates	6.2	2.7	7.4	159	141	147	141	< 50
750	Sulfates	6.6	2.7	6.0	122	105	133	141	883
750	Oxides	6.2	2.5	6.4	130	109	111	134	715
750	Chelates	5.8	2.5	7.3	147	132	141	136	951
750	*Low-sulfates*	5.8	2.4	6.7	54	66	29	136	930

*n. a.* not analyzed

Calculated metabolizable energy concentration: 13.7 and 13.9 MJ/kg DM in starter and grower diets

Calculated crude protein concentration: 248 and 227 g/kg DM in starter and grower diets

The treatments included in the full-factorial design were formulated to contain 100/100/125 mg/kg of Zn/Mn/Cu supplementation

The low-sulfates treatment was formulated to contain 15/40/7.5 mg/kg of Zn/Mn/Cu supplementation as sulfates

### Experimental Procedures

For the determination of average daily feed intake (ADFI), average daily gain (ADG), and feed conversion ratio (FCR), broilers and feed were weighed on a pen basis on days 0, 12, and 28. Calculations were corrected for mortality by accounting for bird days. On day 28, birds were euthanized by carbon dioxide exposure. Ileal digesta was collected from 8 birds per pen by flushing with distilled water, pooled on a pen basis, and immediately put on dry ice. The ileum was defined as the distal two thirds of the segment between Meckel’s diverticulum and 2-cm anterior to the ileo-ceco-colonic junction. The left foot and tibiotarsus (tibia) were collected from 2 randomly selected birds per pen and frozen until further processing. Jejunal tissue (middle section), *pectoralis* muscle tissue, and liver tissue were collected from one randomly selected bird per pen for gene expression analysis. The tissues for gene expression analysis were snap-frozen in liquid nitrogen and stored at − 80°C until further processing.

### Chemical Analyses

Ileal digesta was freeze-dried (Labconco 780601010, Labconco Corporation, Kansas City, MO, USA) and pulverized. Feed and freeze-dried ileal digesta samples were oven-dried at 100°C for 24 h following AOAC Method 934.01 [14] to determine dry matter (DM). For mineral analysis, samples of feed and ileal digesta were ashed in a muffle furnace (6 h, 550°C) with subsequent acid digestion (25% HCl) of the ash using AOAC Method 968.08 [14] and were analyzed for minerals following EPA Method 200.7 [15] by inductively coupled plasma-optical emission spectroscopy (ICP-OES, Spectro Arcos FHS16, Germany). Titanium was determined as described by Short et al. [16]. In brief, ashed samples were digested with 7.4 M sulfuric acid, hydrogen peroxide (30%) was added, and absorbance was measured in a spectrophotometer (Spectronic 200, Thermo Scientific, Waltham, MA, US) at 410 nm.

Tibiae were thawed and adhering soft tissue, fibula bones, and cartilage caps were manually removed. At the joint (*articulatio intertarsalis*), feet were detached and used completely below the joint including tissues, claws, and skins. Tibiae and feet were cleaned with distilled water, carefully patted, and dried for 48 h (tibiae) and 72 h (feet) at 100°C in a convection oven (VWR International, Radnor, PA, USA). Subsequently, the bone samples were cooled in a desiccator and weighed. The dried bones were ashed for 24 h (tibiae) or 48 h (feet) at 600°C in a muffle furnace (Lindenberg/Blue M, Thermo Scientific, Waltham, MA, USA). Ashed tibiae and feet were weighed and ground to 0.5 mm (Retsch, ZM 200, Haan, Germany). Minerals analysis of tibia ash was conducted as described for feed and ileal digesta.

The InsP_3-6_ isomers were determined in feed and ileal digesta using the method of Zeller et al. [17] with slight modifications described by Sommerfeld et al. [18]. To determine *myo*-inositol concentrations, digesta and feed were processed, and measurements were performed using an Agilent 5977A gas chromatograph/mass spectrometer (Waldbronn, Germany) as described in Sommerfeld et al. [19].

An ELISA assay was used to determine phytase activity in feed samples (AB Vista, Plantation, FL, USA), and results were subsequently converted to FTU per kilogram.

### Quantitative Real-Time PCR Analysis

Quantitative real-time PCR was used to analyze gene expression of selected intestinal mineral transporters and protein synthesis and degradation in *pectoralis* muscle and liver tissue. Tissues (approximately 3 mm × 3 mm) were homogenized in QIAzol®Lysis Reagent (QIAGEN, Hilden, Germany). Total RNA was extracted according to the manufacturer’s instructions. Before converting extracted RNA to cDNA in a 96-well PCR system (Applied Biosystems™ Veriti™ Thermal Cycler) using a high-capacity cDNA reverse transcription kit (Thermo Fisher Scientific, Waltham, MA, USA), RNA quantity and quality were measured by use of synergy HTX multi-mode reader (BioTek Instruments, Winooski, VT, USA) and diluted accordingly. After cDNA conversion, the cDNA was diluted, and by using reaction master mix iTaq Universal SYBR® Green Supermix (Bio-Rad Laboratories, Hercules, CA, USA) real-time PCR reaction was performed in a CFX96™ Real-Time System (Bio-Rad Laboratories, Hercules, CA, USA). The PCR conditions were as follows: 95°C for 5 min; 40 cycles of 95°C for 10 s, annealing temperature for 45 s. The annealing temperature was adapted according to the primer. All samples were run in duplicate, and the 2^(−ΔΔCt)^ method [20] was used for data analysis. The low-sulfates treatment was used as a control for the ^ΔΔ^Ct normalization. All primers and their accession numbers are shown in Table 4.

**Table 4:** Primers with GenBank accession number, function, sequence of forward and reverse primer

Gene symbol	Full name	Accession number	Function	Forward primer	Reverse primer
*GAPDH*	*Glyceraldehyde-3-phosphate dehydrogenase*	NM_204305.2	Housekeeping gene	CTTTGGCATTGTGGAGGGTC	ACGCTGGGATGATGTTCTGG
*CTR1 (SLC31A1)*	*Copper transporter 1*	XM_046929029.1	Cu transport	CATCTTCAGGAGGTGGTCAT	CAACGGCACATTCTCATAGC
*ZnT5 (SLC30A5)*	*Zinc transporter 5*	NM_001031419.3	Zn transport	TGGAGGACCAGCCAAGACAA	TCCCTCAGGATGTTCTGCTATTTT
*ZnT10 (SLC30A10)*	*Zinc transporter 10*	XM_040668721.2	Zn transport	ATCAGATGGCACAAGGCAAACA	GAACAGACCTACGATCCCGACA
*NaPi type IIb (SLC34A2)*	*Na-dependent phosphate transport protein type IIb*	AY389468.2	Phosphate transport	GGAAGCATTGCTGCGGATT	GCACCCTCCTGTTCTGCATT
*PiT-2 (SLC20A2)*	*Na-dependent phosphate transporter 2*	NM_001305398.2	Phosphate transport	GCTGGGAGCAAAAGTAGGAGA	AAACAGCAGAACCAACCATCG
*mTOR*	*Mechanistic target of rapamycin*	XM_040689168.2	Protein synthesis	GCTGTGATGTCAATGGTTGG	CTCTTGTCATCGCAACCTCA
*S6K*	*S6Kinase*	XM_046931821.1	Protein synthesis	AGTGGCCTGCAGATAGCAGT	TGGGTCAGCCCTTCTACAAC
*EIF4EBP1*	*Eukaryotic translation initiation factor 4E binding protein 1*	XM_040689367.2	Protein synthesis	TGAGTGCCTTCCTGTTTCCT	TATTCACACCCACACGGAGA
*PRKAB2*	*Protein kinase AMP-activated non-catalytic subunit beta 2*	XM_046922944.1	Protein degradation	CCATCCTGCTGTCCCATTAT	CCTGTCAGGAGCAAGGAAAG
*FBXO32*	*F-box only protein 32*	XR_006933395.1	Protein degradation	GTGTTGTTCTGCCCATGTTG	CACTGGAGGAAGACGGGATA
*TRIM63*	*Tripartite motif containing 63*	XM_040689676.2	Protein degradation	AGGAGCTCTGTGCACGTTTT	CTTCTCCAGCTGCTCCTTGT

### Calculations and Statistical Analysis

On a pen basis, growth performance (ADG, ADFI, FCR) was calculated for the entire trial duration (day 0 to 28) and was corrected for mortality. Digesta samples and bones were pooled on a pen basis, and the pen was considered the experimental unit. The bird was considered the experimental unit for gene expression as tissues were obtained from individual birds.

The prececal InsP_6_ disappearance and prececal P and Ca digestibility were calculated based on analyzed concentrations of Ti, P, Ca, and InsP_6_ in feed and digesta, using the following equation:

$${\text{y}}\left(X\right)=100-\left\{100\times \left(\frac{\text{Ti in feed}}{\text{Ti in digesta}}\times \frac{{\text{X}}\text{ in digesta}}{{\text{X}}\text{ in feed}}\right)\right\}$$

where ${\text{y}}\left({\text{X}}\right)$ is the disappearance or digestibility of ${\text{X}}$ in % and ${\text{X}}$ is the concentration of P, Ca, or InsP_6_ in feed and digesta.

Data were checked with ANOVA residual diagnostic plots for normal distribution and variance homogeneity and analyzed in a nested 2-factorial analysis of variance using the GLIMMIX procedure of the software package SAS (version 9.4, SAS Institute Inc., Cary, NC); means were separated using *t* test. The following model was chosen:

$${\text{y}}_{\text{ijkl}}\text{=}\mu {{\text{+}{\text{a}}}_{\text{i}}\text{+}{\text{b}}}_{\text{j(i)}}\text{+}{\text{c}}_{\text{k(i)}}\text{+(}{\text{bc)}}_{{\text{jk}}\left({\text{i}}\right)}\text{+}{\delta }_{\text{l}}\text{+}{\varepsilon }_{\text{ijkl}}$$

where ${\text{y}}_{\text{ijkl}}$ is the response variable, $\mu$ is the overall mean, ${\text{a}}$ represents the fixed effect of the *i*th treatment group (${\text{i}}$ = low-sulfates or others), ${\text{b}}$ represents the fixed effect of the *j*th phytase supplementation level within the $\textit{i}$th treatment group (${\text{j}}$ = 0 or 750 FTU/kg), ${\text{c}}$ represents the fixed effect of the *k*th trace mineral source within the $\textit{i}$th treatment group (${\text{k}}$ = sulfates or oxides or chelates), ${\text{bc}}$ is the corresponding interaction of the *j*th phytase supplementation level and the *k*th trace mineral source within the *i*th treatment group, $\delta$ is the random effect of *l*th block (${\text{l}}$ = 1–8), and ${\varepsilon }_{\text{ijkl}}$ is the residual error. Statistical significance was declared at *P* ≤ 0.050.

## Results

### Ileal InsP_x_ and *myo*-inositol concentrations

Ileal concentrations of InsP_6_ were affected by PHY × TMS interaction (*P* = 0.047, Table 5). The diets without phytase supplementation containing sulfates or oxides had higher ileal InsP_6_ concentrations than the diets containing chelates. Phytase supplementation decreased ileal InsP_6_ concentrations to a similar level regardless of TMS used. The Ins(1,2,4,5,6)P_5_ concentrations in sulfate diets were higher than in chelate diets (*P* < 0.021). Phytase supplementation increased Ins(1,2,3,4,5)P_5_ concentration (*P* < 0.001). The Ins(1,2,3,4,6)P_5_ isomer occurred at a quantifiable level only in diets without exogenous phytase. The concentrations of Ins(1,2,5,6)P_4_ and Ins(1,2,3,4)P_4_ were higher in diets with exogenous phytase than without (*P* < 0.001). Phytase supplementation increased InsP_3x_ concentrations (*P* < 0.001). The concentrations of InsP_3x_ were highest in birds fed the low-sulfates diet (*P* < 0.001). The PHY × TMS interaction affected ileal MI concentrations (*P* = 0.050). Ileal MI concentrations were higher in diets with exogenous phytase than without. Without exogenous phytase, feeding oxides resulted in a higher MI concentration than sulfates, whereas with exogenous phytase, feeding sulfates resulted in a higher MI concentration than feeding chelates.

**Table 5:** Effect of phytase supplementation and trace mineral source (TMS)^1^ on ileal InsP_x_ and *myo*-inositol concentrations (μmol/g dry material) in broiler chickens fed experimental diets from day 0 to 28

Treatment		InsP_6_	Ins(1,2,4,5,6)P_5_	Ins(1,2,3,4,5)P_5_	Ins(1,2,3,4,6)P_5_	Ins(1,2,5,6)P_4_	Ins(1,2,3,4)P_4_	InsP_3x_^2^	*Myo*-inositol
Phytase, FTU/kg	TMS								
0	Sulfates	35.4^a^	1.1	1.3	0.6	< LOQ	0.4	0.9	7.6^ed^
0	Oxides	34.0^a^	0.9	1.4	0.7	< LOQ	0.4	0.9	9.4^d^
0	Chelates	30.3^b^	0.9	1.7	0.6	0.3	0.4	0.8	6.1^e^
750	Sulfates	14.5^ cd^	1.1	2.9	< LOQ^3^	2.5	0.6	2.0	17.2^a^
750	Oxides	17.3^c^	1.1	3.1	< LOQ	2.2	0.6	1.8	15.4^ab^
750	Chelates	15.4^c^	0.8	2.6	< LOQ	2.1	0.7	2.1	14.2^b^
750	*Low-sulfates*^4^	11.9^d^	0.9	2.4	< LOQ	3.6	0.4	2.8	12.1^c^
Pooled SEM		1.21	0.08	0.20			0.06	0.16	0.72
Low-sulfates		11.9^b^	0.9	2.4			0.4	2.8^a^	12.1
Treatments^5^		24.5^a^	1.0	2.2			0.5	1.4^b^	11.6
Pooled SEM		0.59	0.05	0.11			0.04	0.09	0.36
Main effects									
Phytase	0	33.2	1.0	1.5^b^			0.4^b^	0.9^c^	7.7
	750	15.7	1.0	2.9^a^			0.6^a^	2.0^b^	15.6
Pooled SEM		0.70	0.06	0.13			0.04	0.10	0.42
TMS	Sulfates	25.0	1.1^a^	2.1			0.5	1.5	12.4
	Oxides	25.7	1.0^ab^	2.2			0.5	1.4	12.4
	Chelates	22.9	0.9^b^	2.1			0.5	1.5	10.1
Pooled SEM		0.85	0.06	0.15			0.05	0.11	0.51
P-values									
Low-sulfates vs. treatments^6^		< 0.001	0.521	0.175			0.189	< 0.001	0.571
TMS		0.065	0.021	0.837			0.746	0.739	0.003
Phytase		< 0.001	1.000	< 0.001			< 0.001	< 0.001	< 0.001
TMS × phytase		0.047	0.256	0.074			0.968	0.476	0.050

Data are given as least square means; *n* = 8 pens

Means within a column not showing a common superscript differ (*P* ≤ 0.050)

^1^The treatments included in the full-factorial design were formulated to contain 100/100/125 mg/kg of Zn/Mn/Cu supplementation

^2^At least one of the following isomers: Ins(1,2,6)P_3_, Ins(1,4,5)P_3_, and Ins(2,4,5)P_3_

^3^ < LOQ: not quantifiable in the majority of samples, limit of quantification of Ins(1,2,3,4,6)P_5_ and Ins(1,2,5,6)P_4_: 0.3 μmol/g

^4^The low-sulfates treatment was formulated to contain 15/40/7.5 mg/kg of Zn/Mn/Cu supplementation as sulfates

^5^Estimate for all treatments included in the full-factorial design (does not include low-sulfates treatment)

^6^Compares low-sulfates treatment with estimate for all other treatments

### Prececal InsP_6_ Disappearance and Mineral Digestibility

Prececal InsP_6_ disappearance was affected by PHY × TMS interaction (*P* < 0.001, Table 6). In the absence of exogenous phytase, birds fed chelates showed a higher prececal InsP_6_ disappearance than those fed oxides or sulfates. InsP_6_ disappearance was increased by phytase supplementation, without differences among the TMS. The InsP_6_ disappearance was similar between birds receiving the low-sulfates diet and those receiving the PHY+ diets. The PHY × TMS interaction affected prececal P digestibility (*P* = 0.003). Whereas the prececal digestibility of P in diets without exogenous phytase varied depending on TMS used, the TMS had no effect on prececal P digestibility in phytase-supplemented diets. Prececal P digestibility of birds fed the low-sulfates diet was lower than for birds receiving other diets (*P* < 0.001). Prececal digestibility of Ca was influenced by PHY × TMS interaction (*P* = 0.009). Whereas birds fed sulfates or chelates had similar prececal Ca digestibility whether phytase was supplemented or not, birds fed oxides had higher Ca digestibility in phytase-supplemented diets. Birds receiving the low-sulfate diet had lower prececal Ca digestibility than birds receiving other diets (*P* < 0.001).

**Table 6:** Effect of phytase supplementation and trace mineral source (TMS)^1^ on prececal InsP_6_ disappearance and mineral digestibility (%) in broiler chickens fed experimental diets from day 0 to 28

Treatment		InsP_6_ disappearance	P digestibility	Ca digestibility
Phytase, FTU/kg	TMS			
0	Sulfates	21.1^c^	56.7^c^	60.4^ab^
0	Oxides	22.7^c^	55.4^c^	53.7^c^
0	Chelates	33.8^b^	65.2^b^	56.0^bc^
750	Sulfates	71.8^a^	72.5^a^	58.5^ab^
750	Oxides	66.3^a^	73.3^a^	61.8^a^
750	Chelates	66.1^a^	71.8^a^	56.9^bc^
750	*Low-sulfates*^2^	69.4^a^	51.4^c^	27.2^d^
Pooled SEM		2.24	3.12	2.49
Low-sulfates		69.4^a^	51.4^b^	27.2^b^
Treatments^3^		47.0^b^	65.8^a^	57.9^a^
Pooled SEM		1.08	2.77	2.07
Main effects				
Phytase	0	25.9	59.1	56.7
	750	68.1	72.5	59.1
Pooled SEM		1.29	2.80	2.12
TMS	Sulfates	46.4	64.6	59.4
	Oxides	44.5	64.3	57.7
	Chelates	50.0	68.5	56.4
Pooled SEM		1.58	2.88	2.21
P-values				
Low-sulfates vs. treatments^4^		< 0.001	< 0.001	< 0.001
TMS		0.056	0.027	0.175
Phytase		< 0.001	< 0.001	0.071
TMS × phytase		< 0.001	0.003	0.009

Data are given as least square means; *n* = 8 pens

Means within a column not showing a common superscript differ (*P* ≤ 0.050)

^1^The treatments included in the full-factorial design were formulated to contain 100/100/125 mg/kg of Zn/Mn/Cu supplementation

^2^The low-sulfates treatment was formulated to contain 750 FTU/kg phytase and 15/40/7.5 mg/kg of Zn/Mn/Cu supplementation as sulfates

^3^Estimate for all treatments included in the full-factorial design (does not include low-sulfates treatment)

^4^Compares low-sulfates treatment with estimate for all other treatments

### Bone Ash

Phytase supplementation increased tibia and foot ash quantity and concentration (*P* < 0.050, Table 7). Tibia ash concentration was higher when birds received the low-sulfate diet compared with the other diets (*P* = 0.007). Foot ash concentration was higher in birds receiving chelates than sulfates (*P* = 0.038). Diet did not affect concentrations of P, Ca, and Cu in tibia ash. Interaction of PHY × TMS affected Zn concentration in tibia ash (*P* = 0.034). The concentration of Zn was increased in sulfate and oxide diets by phytase supplementation but remained at similar and low levels in diets with chelates, whether phytase was supplemented or not. The Mn concentration in tibia ash was lower in birds receiving the low-sulfate diet than in birds receiving the other diets and was increased by phytase supplementation (*P* < 0.001).

**Table 7:** Effect of phytase supplementation and trace mineral source (TMS)^1^ on bone mineralization in broiler chickens fed experimental diets from day 0 to 28

						Minerals in tibia ash				
Treatment		Foot ash^2^		Tibia ash^2^		P	Ca	Zn	Mn	Cu
		g/bone	g/g	g/bone	g/g	g/kg	g/kg	mg/kg	mg/kg	mg/kg
Phytase, FTU/kg	TMS									
0	Sulfates	1.623	0.146	1.669	0.467	159	305	290^c^	7.6	2.6
0	Oxides	1.736	0.155	1.774	0.475	158	306	281^ cd^	7.3	3.1
0	Chelates	1.802	0.157	1.755	0.482	160	308	273^d^	7.3	3.2
750	Sulfates	1.822	0.158	1.901	0.488	157	302	309^a^	8.6	2.8
750	Oxides	1.907	0.156	1.924	0.486	159	305	307^ab^	9.2	2.8
750	Chelates	1.837	0.161	1.795	0.489	160	306	270^d^	8.9	2.9
750	*Low-sulfates*^3^	1.864	0.160	1.948	0.495	159	305	292^bc^	6.1	2.5
Pooled SEM		0.070	0.003	0.074	0.005	2.16	3.41	6.05	0.43	0.28
Low-sulfates		1.864	0.160	1.948	0.495^a^	159	305	292	6.1^b^	2.5
Treatments^4^		1.788	0.156	1.803	0.481^b^	159	305	288	8.1^a^	2.9
Pooled SEM		0.035	0.001	0.036	0.002	1.54	2.20	3.46	0.21	0.18
Main effects										
Phytase	0	1.720^b^	0.153^b^	1.732^b^	0.475^b^	159	306	281	7.4^b^	3.0
	750	1.855^a^	0.159^a^	1.873^a^	0.488^a^	159	304	295	8.9^a^	2.8
Pooled SEM		0.041	0.002	0.043	0.003	1.61	2.37	3.86	0.25	0.20
TMS	Sulfates	1.722	0.152^b^	1.785	0.478	158	303	299	8.1	2.7
	Oxides	1.821	0.155^ab^	1.849	0.480	158	305	294	8.2	3.0
	Chelates	1.820	0.159^a^	1.775	0.486	160	307	271	8.1	3.1
Pooled SEM		0.050	0.002	0.052	0.003	1.76	2.66	4.51	0.30	0.22
P-values										
Low-sulfates vs. treatments^5^		0.315	0.126	0.074	0.007	0.939	0.843	0.547	< 0.001	0.125
TMS		0.277	0.038	0.559	0.222	0.523	0.558	< 0.001	0.933	0.361
Phytase		0.022	0.010	0.024	0.001	0.977	0.355	0.004	< 0.001	0.389
TMS × phytase		0.459	0.094	0.436	0.313	0.795	0.900	0.034	0.528	0.427

Data are given as least square means; *n* = 8 pens

Means within a column not showing a common superscript differ (*P* ≤ 0.050)

^1^The treatments included in the full-factorial design were formulated to contain 100/100/125 mg/kg of Zn/Mn/Cu supplementation

^2^Dry matter basis

^3^The low-sulfates treatment was formulated to contain 750 FTU/kg phytase and 15/40/7.5 mg/kg of Zn/Mn/Cu supplementation as sulfates

^4^Estimate for all treatments included in the full-factorial design (does not include low-sulfates treatment)

^5^Compares low-sulfates treatment with estimate for all other treatments

### Gene Expression

#### Jejunum

Gene expression of *Zn transporter 5* (*ZnT5*) in the jejunum was affected by PHY × TMS interaction (*P* = 0.024, Table 8). Whereas the expression of *ZnT5* remained at similar levels in diets with oxides and chelated TM whether phytase was supplemented or not, the expression was increased in sulfate diets when phytase was supplemented. Gene expression of *Zn transporter 10* (*ZnT10*) and of phosphate transporter (*NaPi-IIb*) was not affected by diet (*P* > 0.050). Expression of phosphate transporter (*PiT-2*) tended to be affected by phytase supplementation (*P* = 0.080). The gene expression of *Cu transporter 1* (*CTR1*) tended to be affected by TMS (*P* = 0.058).

**Table 8:** Effect of phytase supplementation and trace mineral source (TMS)^1^ on gene expression of mineral transporters in the jejunum of broiler chickens fed experimental diets from day 0 to 28

Treatment		*ZnT5*	*ZnT10*	*PiT-2*	*NaPi-IIb*	*CTR1*
Phytase, FTU/kg	TMS					
0	Sulfates	0.963^b^	1.472	0.990	0.854	0.974
0	Oxides	1.253^ab^	2.082	1.212	1.248	1.198
0	Chelates	1.126^b^	1.087	1.215	0.847	1.422
750	Sulfates	1.607^a^	2.197	1.407	0.963	1.157
750	Oxides	1.206^ab^	1.914	1.226	1.000	1.060
750	Chelates	0.955^b^	1.632	1.472	0.984	1.230
750	*Low-sulfates*^2^	0.994^b^	1.000	1.002	1.000	1.002
Pooled SEM		0.1625	0.3690	0.1521	0.1971	0.1090
Low-sulfates		0.994	1.000	1.002	1.000	1.002
Treatments^3^		1.185	1.731	1.254	0.983	1.173
Pooled SEM		0.0978	0.1767	0.0742	0.0964	0.0532
Main effects						
Phytase	0	1.114	1.547	1.139	0.983	1.198
	750	1.256	1.914	1.368	0.982	1.149
Pooled SEM		0.1085	0.2128	0.0889	0.1155	0.0636
TMS	Sulfates	1.285	1.834	1.198	0.909	1.065
	Oxides	1.229	1.998	1.219	1.124	1.129
	Chelates	1.040	1.359	1.343	0.915	1.326
Pooled SEM		0.1237	0.2598	0.1085	0.1412	0.0777
P-values						
Low-sulfates vs. treatments^4^		0.239	0.090	0.127	0.933	0.146
TMS		0.238	0.210	0.586	0.493	0.058
Phytase		0.269	0.235	0.080	0.998	0.591
TMS × phytase		0.024	0.470	0.450	0.578	0.201

Data are given as least square means; *n* = 6

Means within a column not showing a common superscript differ (*P* ≤ 0.050)

^1^The treatments included in the full-factorial design were formulated to contain 100/100/125 mg/kg of Zn/Mn/Cu supplementation

^2^The low-sulfates treatment was formulated to contain 750 FTU/kg phytase and 15/40/7.5 mg/kg of Zn/Mn/Cu supplementation as sulfates

^3^Estimate for all treatments included in the full-factorial design (does not include low-sulfates treatment)

^4^Compares low-sulfates treatment with estimate for all other treatments

#### Pectoralis Muscle and Liver

In the *pectoralis* muscle, expression of *EIF4EBP1* (*eukaryotic translation initiation factor 4E binding protein 1*) and *mTOR* (*mechanistic target of rapamycin*) was not affected by diet (*P* > 0.050, Table 9). Expression of *Trim63* (*Tripartite motif containing 63*) and *FBXO32* (*F-box only protein 32*) was increased in the low-sulfate diet compared to the other diets (*P* < 0.050).

**Table 9:** Effect of phytase supplementation and trace mineral source (TMS)^1^ on expression of genes related to protein synthesis and degradation in breast muscle and liver in broiler chickens fed experimental diets from day 0 to 28

Treatment		*Pectoralis* muscle				Liver					
Phytase, FTU/kg	TMS	*mTOR*	*EIF4EBP1*	*Trim63*	*FBXO32*	*mTOR*	*S6kinase*	*EIF4EBP1*	*Trim63*	*FBXO32*	*PRKAB2*
0	Sulfates	0.576	0.864	0.255	0.274	1.083	0.956	0.845	0.703	0.750	1.114
0	Oxides	0.532	0.699	0.337	0.385	0.975	0.978	0.974	1.160	1.250	0.994
0	Chelates	0.621	0.851	0.458	0.433	1.028	1.053	0.822	1.233	1.186	1.156
750	Sulfates	0.870	0.971	0.491	0.490	1.081	0.865	1.083	0.878	0.850	1.030
750	Oxides	0.929	0.921	0.556	0.584	1.063	1.112	1.048	0.798	0.752	0.962
750	Chelates	0.912	1.022	0.473	0.481	0.946	0.979	0.980	0.737	0.791	0.834
750	*Low-sulfates*^2^	1.009	1.008	0.986	0.986	1.006	1.015	1.000	1.013	1.010	1.002
Pooled SEM		0.2338	0.1975	0.2078	0.1987	0.0653	0.0989	0.0852	0.1806	0.1766	0.0988
Low-sulfates		1.009	1.008	0.986^a^	0.986^a^	1.006	1.015	1.000	1.013	1.010	1.002
Treatments^3^		0.740	0.888	0.428^b^	0.441^b^	1.029	0.991	0.959	0.918	0.930	1.015
Pooled SEM		0.1239	0.1028	0.1208	0.1141	0.0351	0.0731	0.0413	0.0965	0.0971	0.0486
Main effects											
Phytase	0	0.576	0.805	0.350	0.364	1.029	0.996	0.880^b^	1.032	1.062	1.088
	750	0.904	0.971	0.507	0.518	1.030	0.985	1.037^a^	0.804	0.798	0.942
Pooled SEM		0.1419	0.1187	0.1344	0.1267	0.0403	0.0764	0.0494	0.1109	0.1106	0.0578
TMS	Sulfates	0.723	0.917	0.373	0.382	1.082	0.911	0.964	0.790	0.800	1.072
	Oxides	0.731	0.810	0.446	0.484	1.019	1.045	1.011	0.979	1.001	0.978
	Chelates	0.766	0.937	0.465	0.457	0.987	1.016	0.901	0.985	0.988	0.995
Pooled SEM		0.1689	0.1419	0.1559	0.1475	0.0478	0.0825	0.0605	0.1319	0.1300	0.0704
P-values											
Low-sulfates vs. treatments^4^		0.312	0.594	0.017	0.018	0.732	0.774	0.655	0.618	0.662	0.903
TMS		0.978	0.774	0.882	0.848	0.315	0.209	0.448	0.458	0.421	0.602
Phytase		0.082	0.290	0.323	0.310	0.982	0.872	0.033	0.127	0.070	0.082
TMS × phytase		0.964	0.956	0.813	0.882	0.430	0.309	0.642	0.141	0.189	0.309

Data are given as least square means; *n* = 6

Means within a column not showing a common superscript differ (*P* ≤ 0.050)

^1^The treatments included in the full-factorial design were formulated to contain 100/100/125 mg/kg of Zn/Mn/Cu supplementation

^2^The low-sulfates treatment was formulated to contain 750 FTU/kg phytase and 15/40/7.5 mg/kg of Zn/Mn/Cu supplementation as sulfates

^3^Estimate for all treatments included in the full-factorial design (does not include low-sulfates treatment)

^4^Compares low-sulfates treatment with estimate for all other treatments

In the liver, expression of *mTOR* and *S6kinase* was not affected by diet (*P* > 0.050, Table 9). Supplementation of phytase increased the expression of *EIF4EBP1* (*P* = 0.033). The expression of *Trim63* was not affected by diet (*P* > 0.050). Phytase supplementation tended to affect the expression of *FBXO32* and *PRKAB2* (*P* < 0.100).

### Performance Traits

At the beginning of the experiment (day 0), the average body weight of broiler chickens was 45 g. During the experiment, 43 birds (4.8%) died, but mortality was unrelated to treatment. Birds fed the low-sulfate diet had higher ADG, ADFI, and lower FCR than birds fed other diets (*P* < 0.050, Table 10). Birds fed sulfates or oxides tended to have higher ADG (*P* = 0.057) and had lower FCR (*P* = 0.009) than birds receiving chelates.

**Table 10:** Effect of phytase supplementation and trace mineral source (TMS)^1^ on performance traits in broiler chickens fed the experimental diets from day 0 to 28

Treatment		ADG (g/d)	ADFI (g/d)	FCR (g/g)
Phytase, FTU/kg	TMS			
0	Sulfates	59.5	83.5	1.41
0	Oxides	58.3	81.7	1.40
0	Chelates	55.7	80.7	1.44
750	Sulfates	58.9	83.2	1.41
750	Oxides	61.1	85.2	1.39
750	Chelates	57.7	81.8	1.42
750	*Low-sulfates*^2^	62.7	86.9	1.39
Pooled SEM		1.39	1.76	0.01
Low-sulfates		62.7^a^	86.9^a^	1.39^b^
Treatments^3^		58.5^b^	82.7^b^	1.41^a^
Pooled SEM		0.82	1.06	0.01
Main effects				
Phytase	0	57.8	82.0	1.42
	750	59.2	83.4	1.41
Pooled SEM		0.90	1.17	0.01
TMS	Sulfates	59.2	83.3	1.41^b^
	Oxides	59.7	83.4	1.40^b^
	Chelates	56.7	81.3	1.43^a^
Pooled SEM		1.05	1.34	0.01
P-values				
Low-sulfates vs. treatments^4^		0.004	0.019	0.021
TMS		0.057	0.335	0.009
Phytase		0.189	0.286	0.261
TMS × phytase		0.400	0.478	0.299

Data are given as least square means; *n* = 8 pens

Means within a column not showing a common superscript differ (*P* ≤ 0.050)

^1^The treatments included in the full-factorial design were formulated to contain 100/100/125 mg/kg of Zn/Mn/Cu supplementation

^2^The low-sulfates treatment was formulated to contain 750 FTU/kg phytase and 15/40/7.5 mg/kg of Zn/Mn/Cu supplementation as sulfates

^3^Estimate for all treatments included in the full-factorial design (does not include low-sulfates treatment)

^4^Compares low-sulfates treatment with estimate for all other treatments

## Discussion

The first objective was to investigate how different TMS affect prececal phytate breakdown to MI and the second objective was to investigate whether TMS and exogenous phytase affect trace mineral concentration in the bone.

### Effects of Trace Mineral Source on Phytate Breakdown

The InsP_6_ disappearance was higher for chelates than for oxides or sulfates in the PHY− diets, partially confirming the first hypothesis of this trial that the TMS affects InsP_6_ disappearance. This supports the assumption that chelated trace minerals are less prone to interact with InsP_6_ than inorganic trace minerals [21–23]. This could be because chelates might dissociate slower than sulfates and oxides, leaving less time for the formation of complexes of divalent cations and phytate. Another hypothesis was proposed by Wedekind et al. [23], who suggested that the ligands of chelates may compete with phytate for its ability to bind Zn. In contrast, Schlegel et al. [24] suggested that native (plant-derived) InsP_6_ does not interact with supplemental Zn and that chelation of inorganic Zn sources is not necessary [25]. Differences in the behavior of chelated sources could be a reason why divergent results were found in the literature. Chelated sources differ in their chelation strength, and this determines whether the chelation remains stable in different pH conditions [26].

In PHY+ diets, however, no differences in InsP_6_ disappearance were observed between TMS in the present experiment. This contradicts an in vitro study by Santos et al. [8], which showed that when Na-phytate was used, different sources (for example, glycinates and sulfates) of Zn, Cu, and Mn differed in their inhibitory effect on exogenous phytase efficacy. A possible explanation for these differences could be differences in substrates. The reactivity of Na-phytate and native phytate (derived from corn and soybean in the present trial) is different and could be higher for Na-phytate than native phytate [27]. In addition, the order in which the reactions or interactions of divalent cations and phytate occur may be different in vitro and in vivo. Whereas in vitro all reactions occur at the same time, the interactions in vivo occur in different sections of the gastrointestinal tract to different extents due to varying conditions. Another difference is that in the present study, the effects of Zn, Cu, and Mn were tested in combination, whereas in the in vitro study by Santos et al. [8], individual TM were tested.

A major part of phytate degradation by exogenous phytase occurs in the proximal digestive tract [17] resulting in a lower potential or possibility of strong complex formation of InsP_6_ with minerals in the small intestine, where pH is favorable for stable phytate-mineral-complex formation [28]. The lower interactive potential could be the reason why no differences were observed in the prececal InsP_6_ disappearance between the TMS in the present experiment upon adding exogenous phytase. Although the concentration of partially dephosphorylated InsP_x_ increased in the small intestine in phytase-supplemented diets, the overall interactive potential is reduced because the binding strength between InsP_x_ and (divalent) cations decreases with decreasing degree of phosphorylation [29].

Complete dephosphorylation of InsP_6_ leads to the release of MI. Consistent with previous studies [19, 30, 31], the ileal MI concentration was increased by exogenous phytase in the present study. To our knowledge, there is no information in the literature on the release of MI when different TMS are used. In the present study, ileal MI concentrations differed depending on TMS used: whereas in PHY− diets, the highest MI concentration was observed for oxides, in PHY+ diets, the highest MI concentration was found for sulfates. The supplementation of chelates resulted in the lowest MI concentrations. A significant difference in the ileal MI concentration was also found between the phytase-supplemented sulfate diet and the low-sulfate diet. The lower concentration of MI coincided with a higher accumulation of Ins(1,2,5,6)P_4_ and InsP_3x_ in the low-sulfate diet. This could indicate lower activity of endogenous phosphatases in this diet. The Zn concentration in the low-sulfate diet may not have been sufficient for the endogenous phosphatases to develop their full potential since Zn is a cofactor of phosphatases [32]. This fits with the hypothesis suggested by Philippi et al. [33] that low dietary Zn concentrations may not be sufficient to achieve full activity of endogenous phosphatases. However, it has to be kept in mind that MI absorption by the chicken intestine is barely understood [34], and it cannot be ruled out that TMS affected MI transporters.

As expected, prececal P digestibility was increased by exogenous phytase. Except for the low-sulfate diet, P digestibility followed the same pattern as the InsP_6_ disappearance. Potential reasons why the prececal P digestibility of the low-sulfate diet was on a similar level as the PHY− diets could not be found. The chemical composition of this diet gave no indication as to why the prececal P digestibility was much lower than expected. In part, the low prececal P digestibility could be due to a lower concentration of titanium dioxide in the ileal digesta in the low-sulfates diet than in the other diets (data not shown). Still, the reasons why the titanium dioxide concentration was lower could not be identified. The prececal P digestibility of the low-sulfate diet should be interpreted with caution because it is inconsistent with the bone results.

In birds, active intestinal phosphate transport is mainly performed by Na-dependent phosphate transporters such as the *NaPi-IIb* [35]. In the present study, the expression of *NaPi-IIb* and *PiT-2* was not affected by TMS or exogenous phytase or their interaction. As the investigation of gene expression on mRNA level or protein level does not allow us to conclude about the activity of a transporter, both increased transporter activity and increased paracellular uptake could be the mediators for increased phosphate absorption in PHY+ diets.

Prececal Ca digestibility was similar for chelate and sulfate diets whether or not phytase was supplemented. Yenice et al. [36] observed increased serum Ca concentrations in laying hens fed a chelated compared to an inorganic trace mineral mixture. The authors suggested that using chelated trace minerals reduces the amount of free ions in the small intestine, leaving fewer free ions available to form insoluble complexes. Still, because significant amounts of Ca can be excreted via the urine, the prececal Ca digestibility and serum Ca concentrations are difficult to interpret. Contrary to the hypothesis of Yenice et al. [36], Bertolo et al. [37] hypothesized that Ca^2+^ and Zn^2+^ may use the same non-specific transporter in the brush border membrane, leading to competition between Ca^2+^ and Zn^2+^. Yet, in the present study, Ca digestibility was significantly lower in the low-sulfates diet (15 mg/kg Zn supplementation) than in the sulfate diet (100 mg/kg Zn supplementation).

In consistency with other studies [38, 39], phytase supplementation increased the quantity (g/bone) of tibia and foot ash and concentration (g/g) of tibia and foot ash in the present study. Foot ash concentration was higher in the chelate diets than in the sulfate diets, reflecting the higher prececal InsP_6_ disappearance and P digestibility of the PHY–chelate diet compared to the PHY–sulfate diet. However, the main effect of TMS was not significant for tibia ash. It cannot be excluded that the composition of the soft tissue, which is also included in the ashing of the foot, changes depending on the TMS.

Augspurger et al. [40] found that tibia ash concentration was reduced in broilers when very high concentrations of Zn (800 mg/kg) were added to a phytase-supplemented diet compared with phytase supplementation alone. Mohanna and Nys [41] and Gosh et al. [42] observed no effect of the dietary Mn concentration (40 or 100 mg/kg Mn) on tibia ash quantity or concentration. Furthermore, high dietary Cu concentrations (200 mg/kg Cu) did not result in changes in the quantity or concentration of tibia ash in the study by Augspurger et al. [40]. Also, in a study by Banks et al. [43], high supplementations of Cu (up to 250 mg/kg) as Cu sulfate did not affect tibia ash concentrations. In the present study, no effects of trace mineral concentrations were observed on tibia ash quantity or concentration. However, it should be noted that these comparisons are confounded by differences in study duration, Zn concentration (800 vs. 100 mg), Mn concentration (40 vs. 100 mg), and Cu concentration (200/250 vs. 125 mg) and by the simultaneous supplementation of Zn, Cu, and Mn in the present study as opposed to changes of single minerals.

### Effects of Phytase and Trace Mineral Source on Bone Trace Mineral Concentration and Performance

Zinc concentration in tibia ash was increased by phytase supplementation in sulfate and oxide diets. This has been observed before and is probably due to the release of Zn associated with InsP_6_ degradation [44]. Phytase supplementation did not increase the Zn concentration in tibia ash in the diet with chelated TMS in the present trial. Pang and Applegate [45] found that a high Cu concentration (250 mg/kg) supplemented as Cu chelate reduced Zn solubility in duodenum and jejunum. In contrast, the supplementation of Cu sulfate did not lead to reduced Zn solubility, supporting the results of the present study. Yet, no explanation was provided as to why Cu sulfate had no lowering effect on Zn solubility despite the antagonism between Cu and Zn [46]. Mohanna and Nys [41] observed increased Mn concentrations in tibia ash due to the combined supplementation of Mn sulfate and Zn sulfate. This is consistent with the higher Mn concentration in tibia ash in the sulfate diet compared to the low-sulfate diet. However, unlike in the present trial, phytase supplementation did not affect Mn concentration in tibia ash in a 21-day trial [41]. Singh et al. [47] observed an increasing effect of exogenous phytase on tibia Mn concentration on day 42 but not on day 21. When tibia ash is used as a trait to evaluate P availability, it has been shown to be more sensitive in a 4-week assay compared to a 10-day assay [48]. This could be one reason why differences were observed at different time points. In the present experiment, no differences in Mn concentration of tibia ash attributable to TMS were detected when supplemented at the same level. This confirms the results of Li et al. [49], where differences in the Mn concentration of tibia ash were only found due to different Mn supplementation levels but remained the same whether chelated or inorganic Mn sources were used. Consistent with observations by Nguyen et al. [50] in a study with 35-day-old broilers, Cu concentration in the present experiment was not affected by the dietary Cu concentration or the Cu source used.

In contrast to results from Bortoluzzi et al. [51], no consistent association between Zn supplementation and gene expression level of *ZnT5* was found in the present study. In agreement with Hu et al. [52], the expression of *ZnT10* mRNA level in the present experiment tended to be upwardly expressed in the diets with high TM supplementation compared to the low-sulfates diet. In addition, no differences were observed between chelate and sulfate diets with regards to mRNA level of *ZnT10* in the present study or in the study by Hu et al. [52]. However, Hu et al. [52] observed increased expression of *ZnT10* at the protein level in birds receiving a chelated Zn source compared to birds receiving ZnSO_4_. Of note, Buccitelli and Selbach [53] reviewed mRNA-level and protein-level measurements and concluded that both give valuable insights but have limitations in their interpretability in terms of active biological function of proteins. The gene expression on mRNA-level is only a “construction plan” for protein synthesis, and the protein-level simply shows that the respective mRNA was available and the protein was synthesized. Furthermore, the expression of *CTR1* was analyzed at the mRNA level in the present experiment. Similar as in an experiment by Meng et al. [54], no significant differences were found in the expression of *CTR1* due to TMS in the present trial. However, the comparison in the study by Meng et al. [54] was confounded by different Mn concentrations (100 mg/kg Mn as sulfate vs. 50 mg/kg Mn as chelate). Because *CTR1* can be affected by Cu, Zn, and Mn [54, 55], interpretation is difficult.

The expression of genes related to protein synthesis and degradation in the *pectoralis* muscle and liver was analyzed to investigate whether the observed differences in growth performance correspond to changes in gene expression. Olukosi et al. [56] found an indication that differences in growth performance between Zn- and Cu-hydroxychloride compared with Zn- and Cu-sulfate could be partially explained by differences in mRNA expression of a gene in *pectoralis* muscle related to protein degradation. However, genes in the *pectoralis* muscle and liver analyzed in the present study could not indicate as to why growth performance differed when using different TMS. Among the genes analyzed, only mRNA expression of *EIF4EBP1* in the liver was significantly affected by diet. The higher mRNA expression of this gene in the PHY+ diets compared with PHY− diets coincided with a numerically higher ADG of PHY+ diets.

## Conclusions

In conclusion, the prececal InsP_6_ degradation by endogenous phosphatases (PHY− treatments) was slightly higher when chelates were used compared to sulfates or oxides, partially confirming the first hypothesis that TMS affects prececal InsP_6_ degradation. Adding exogenous phytase (750 FTU/kg) removed the differences between different TMS in InsP_6_ disappearance, probably due to a lower potential for complex formation. Since the vast majority of broiler diets is commonly supplemented with phytase, the choice of TMS is likely negligible in terms of phytate degradation.

The second hypothesis that phytase supplementation affects trace mineral concentration in the bone was confirmed for Zn and Mn but not for Cu. Concentrations of Zn and Mn in tibia ash were increased by exogenous phytase, although the supply was above NRC supply recommendations. The concentration of Cu in tibia ash was not affected by exogenous phytase or diet in general. Future studies should examine tissues other than bone to evaluate Cu accumulation in the bird.

## Data Availability

Data are not publicly available but are available from the corresponding author on reasonable request.
