# Correction: Impact of Trace Mineral Source and Phytase Supplementation on Prececal Phytate Degradation and Mineral Digestibility, Bone Mineralization, and Tissue Gene Expression in Broiler Chickens

**DOI:** 10.1007/s12011-025-04535-y

**Published:** 2025-02-03

**Authors:** Hanna Philippi, Vera Sommerfeld, Alessandra Monteiro, Markus Rodehutscord, Oluyinka A. Olukosi

**Affiliations:** 1 Institute of Animal Science, University of Hohenheim, 70599 Stuttgart, Germany; 2 Animine, 74960 Annecy, France; 3 Department of Poultry Science, University of Georgia, Athens, GA 30602 USA

**Correction: Biological Trace Element Research (2024) 202:5235–5250**

10.1007/s12011-024-04076-w

The published version of this article unfortunately contained a mistake.

The corresponding author should be Markus Rodehutscord (markus.rodehutscord@uni-hohenheim.de) instead of Oluyinka A. Olukosi (oaolukosi@uga.edu).

